# Ability of a Wild *Weissella* Strain to Modify Viscosity of Fermented Milk

**DOI:** 10.3389/fmicb.2019.03086

**Published:** 2020-01-28

**Authors:** Elena Bancalari, Marcello Alinovi, Benedetta Bottari, Augusta Caligiani, Germano Mucchetti, Monica Gatti

**Affiliations:** Department of Food and Drug, University of Parma, Parma, Italy

**Keywords:** *Weissella*, exopolysaccharides, milk fermentation, starter cultures, viscosity

## Abstract

Despite the fact that strains belonging to *Weissella* species have not yet been approved for use as starter culture, recent toxicological studies open new perspectives on their potential employment. The aim of this study was to evaluate the ability of a wild *Weissella minor* (*W*4451) strain to modify milk viscosity compared to *Lactobacillus delbrueckii* subsp. *bulgaricus*, which is commonly used for this purpose in dairy products. To reach this goal, milk viscosity has been evaluated by means of two very different instruments: one rotational viscometer and the Ford cup. Moreover, water holding capacity was evaluated. *W*4451, previously isolated from sourdough, was able to acidify milk, to produce polysaccharides *in situ* and thus improve milk viscosity. The ability of *W*4451 to produce at the same time lactic acid and high amounts of polysaccharides makes it a good candidate to improve the composition of starters for dairy products. Ford cup turned out to be a simple method to measure fermented milk viscosity by small- or medium-sized dairies.

## Introduction

*Weissella* is a lactic acid bacteria (LAB) frequently isolated from spontaneous fermented foods and is known to have an important role in the development of their particular features (Fessard and Remize, 2017). Nowadays, *Weissella* species have not yet been approved for commercial use as starter cultures in fermented foods in the United States nor the European Union (Sturino, 2018). However, recent toxicological studies confirmed *Weissella confusa* to be safe (Cupi and Elvig-Jorgensen, 2019), and *Weissella minor* (Collins et al., 1993) strains have been classified as BSL1 (U.S. Department of Health and Human Services, 2009), i.e., low-risk microbes that pose little to no threat of infection in healthy adults. This opens new perspectives in the use of *Weissella* species as starters in fermented foods. Indeed, *Weissella* species have already been used in the production of a variety of fermented foods and beverages and also as probiotics (Kang et al., 2011; Lee et al., 2012; Gomathi et al., 2014; Zhang et al., 2014). Recently, Kariyawasam et al. (2019) used *Weissella* as adjunct starter in cottage cheese.

Furthermore, several *Weissella* species are being extensively studied for their ability to produce significant amounts of extracellular polysaccharides (EPS), which can be used as prebiotics or in food industries with different technological applications (Abriouel et al., 2015).

The ability to produce EPS is a common technological feature among LAB, and it may provide thickening, stabilizing, and water-binding effects in fermented foods (Ruas-Madiedo et al., 2002; Zannini et al., 2015; Sanlibaba and Çakmak, 2016). EPS are long chain molecules with a heterogeneous composition and structure, consisting of either branched or not, repeating units of sugars, or sugars derivatives, that exhibit a broad range of physicochemical properties (Malang et al., 2015; Galle and Arendt, 2014). They can be classified according to their chemical composition and biosynthesis mechanism into two main groups: homopolysaccharides that contain one neutral monosaccharide type and heteropolysaccharides that consist of three to eight monosaccharides, derivatives of monosaccharides, or substituted monosaccharides (HePS) (Torino et al., 2015; Oleksy and Klewicka, 2016). The molecular weight of EPS is high, with HePS ranging from 10^4^ to 10^6^ Da, which is generally lower than the average molecular mass of homopolysaccharides, which ranges up to ∼10^7^ Da (Sanlibaba and Çakmak, 2016; Oleksy and Klewicka, 2016).

Application examples of EPS-producing LAB have been recorded for the production of yogurts, fermented milks, beverages, and fresh cheeses to avoid the addition of milk solids and/or concentrate milk, improve the viscosity, texture, stability, and mouthfeel of the final product, as well as to reduce whey syneresis in the coagulated curd after fermentation or upon storage (Torino et al., 2015).

The application of *Weissella* spp. is quite recent in dairy products (Kimoto-Nira et al., 2011; Lynch et al., 2014), and its ability to produce at the same time lactic acid and high amounts of EPS makes *Weissella* a good candidate to be included in multispecies culture starters also for dairy beverages and/or fresh cheeses.

On the contrary, among the commonly used LAB as dairy starters, *Lactobacillus delbrueckii* subsp. *bulgaricus* has been extensively exploited for the production of EPS in dairy products (Aslim et al., 2005; Schiraldi et al., 2006). In this context, the aim of this study was to evaluate the ability of a wild *W. minor* strain to increase milk viscosity compared to two *Ld. bulgaricus* strains.

## Materials and Methods

### Strains

*Weissella minor*, *W*4451 EPS+, *Ld. bulgaricus*, *Ldb*2214 EPS+, and *Lactococcus lactis Ll* 220 EPS−, belonging to the collection of the Food Microbiology Laboratory (Department of Food and Drug, University of Parma, Italy), and *Ld. bulgaricus*, *Ldb*147 EPS+, kindly provided by Sacco Srl (Cadorago, Italy), were used. *W*4451, was isolated by our group from sourdough and identified by mean of 16s rRNA sequencing analysis (Macrogen Europe, Amsterdam, Netherlands).

The strains were maintained at −80°C as frozen stock cultures in de Man, Rogosa, and Sharpe broth for *Weissella* and *Lactobacillus* strains (Oxoid Ltd., Basingstoke, United Kingdom) and M17 (Oxoid Ltd.) broth for *Lactococcus* strain, containing 20% (*v*/*v*) glycerol. They were recovered by anaerobic incubation in the same media, by two overnight subculturing (2% *v*/*v*) at 37°C for *Weissella* and *Lactobacillus* and 30°C for *Lactococcus*.

### EPS Characterization by Gas Chromatography–Mass Spectrometry

The three EPS + strains (*W*4451, *Ldb*2214, and *Ldb*147) were cultivated in semidefined medium (SDM) [Tween 80 (1 ml/L), ammonium citrate (2 g/L), sodium acetate (5 g/L), MgSO_4_.7H_2_O (0.1 g/L), MnSO_4_ (0.05 g/L), K_2_HPO_4_ (2 g/L), yeast nitrogen base (5 g/L), casein digest (10 g/L)]. It is useful to provide minimal interference with the assays used to characterize EPS (Kimmel and Roberts, 1998). SDM was autoclaved for 15 min at 121°C, and the lactose (Merck, Darmstadt, Germany) aqueous solution (60 g/L), used as carbohydrate source, was sterilized separately (15 min at 121°C) and aseptically added to the medium. The inoculated (2% *v*/*v*) broths were incubated at 37°C for *Lactobacillus* and *Weissella* (Nguyen et al., 2014) and 30°C for *Lc. lactis.* Total EPS from the SDM medium were isolated by means of double cold ethanol precipitation method without dialysis (Rimada and Abraham, 2003). The absence of protein and nucleic acids in the EPS was assessed by spectrophotometric analysis using a UV–visible spectrophotometer (Jasco V-530), at a wavelength of 260–280 nm (Liu et al., 2016).

Recovered EPS were hydrolyzed with 3 ml 2 M trifluoracetic acid under nitrogen for 60 min at 121°C. To the hydrolyzed mixture, 1 ml phenyl-beta-D-glucopyranoside (500 ppm) was added as internal standard. The samples were clarified by syringe filtration on nylon filters (40 μm), and the filtrate evaporated to dryness with nitrogen. Sugars were silylated for 60 min at 60°C adding 600 μl *N*-*N*-dimethylformamide and 200 μl *N*,*O*-bis (trimethylsilyl) trifluoroacetamide containing 1% trimethylchlorosilane. The gas chromatography–mass spectrometry (GC-MS) analysis was carried out using apolar capillary column (HP-5MS, Hewlett Packard, Palo Alto, United States) in the conditions previously reported (Müller-Maatsch et al., 2016).

gas chromatography–mass spectrometry analyses were performed in duplicate. Identification was performed by comparison with retention times and mass spectra of sugars standards (mannose, xylose, ribose, glucose, galactose, rhamnose, arabinose, and fucose). Quantification was performed by means of internal standard, and results were finally expressed as relative percentages.

### Milk Fermentation

Three EPS + strains (*W*4451, *Ldb*2214, and *Ldb*147) were used to perform two subculturing (2% *v*/*v*) at 37°C in skim milk powder (SSM) (Oxoid, Ltd.) reconstituted to 9% (*w*/*v*) and sterilized at 110°C for 15 min. EPS- strain (*Ll* 220) was used as negative control to perform two subculturing (2% *v*/*v*) at 30°C in SSM. Two percent of the last overnight subcultures were used for batch fermentation, which was performed in a bioreactor (working volume, 0.5 L; Applicon Biotechnology, Schiedam, Netherlands), which was sterilized at 121°C for 15 min.

The fermenter was equipped with software BioXpert Lite (Applikon Biotechnology^®^) for system control and data acquisition. The temperature was constantly recorded by Pt-100 sensor (Applikon Biotechnology^®^) and pH by means of Applisensor pH gel sensor (Applikon Biotechnology^®^).

The fermentation was carried out at 37°C for *Lactobacillus* and *Weissella* and 30°C for *Lc. lactis* and was stopped when the pH reached the value of 4.5 by rapid cooling of fermented milk up to 4°C.

### Viscosity Measurement of Fermented Milk

At the end of fermentation, the coagula were broken by means of a rod stirrer (DLH VELP Scientifica^®^) at 210 rpm for 5 min, and then they were maintained at 4°C for 24 h.

Afterwards, the fermented milk samples (100 ml) were equilibrated at 25°C for 90 min and again stirred at 80 rpm for 5 min to reduce possible structural differences among samples caused by the fermentation procedures. After the equilibration time, which helps to reorganize the matrix structure solely due to the chemical characteristics of the sample (Purwandari et al., 2007), the viscosity was measured by means of a Brookfield^®^ DV-I Prime rotational viscometer (Middleboro, MA, United States) equipped with a SC4-18 spindle. The temperature was maintained at 25.0 ± 0.5°C by connecting the small sample adapter chamber (Middleboro, MA, United States) to a thermostatic water bath. The viscometer was connected to a computer for the data acquisition at 1-s intervals in an ASCII table. Apparent viscosity ($\eta =\sigma /\dot{\gamma }$) was measured between the range of shear rate from 0.792 to 7.920 s^–1^. The flow behavior of the samples was described by the Ostwald de Waele model (power law), Equation (1):

$$\sigma =K\times {\left(\gamma \right)}^{n}$$

where σ is the shear stress (Pa), *K* is the consistency index (Pa⋅s*^*n*^*), $\dot{\gamma }$ is the shear rate (s^–1^), and *n* is the flow behavior index.

Moreover, the viscosity values were also compared among the different samples at a shear rate value of 3.96 s^–1^ (η_3__.__96_), which was the central point of the shear rate intervals evaluated.

The viscosity of the fermented milk samples was also measured by means of a brass Ford flow cup (Sacco Srl, Cadorago, Italy), with a capacity of 100 ml and an outflow opening diameter of 3 mm. Measurements were performed according to the standard ISO 2431 (International Standard Organization [ISO], 2019), with the following slight modifications. Because of the high viscosities showed by the fermented milk samples, it was decided to express Ford cup viscosity as gram of sample eluted in 1 min, instead of the time necessary for the elution of the total volume (100 ml). For the measurement, the Ford cup was filled with 100 ml of fermented milk, previously equilibrated at 25°C.

### Determination of Water Holding Capacity of Fermented Milk

Water holding capacity (WHC) may be defined as the ability of the fermented milk to hold water. WHC of all the fermented milk samples at pH 4.5 were determined by the centrifugation method described by Li et al. (2014). Twenty grams of each fermented milk were centrifuged at 2,600 rpm for 15 min. The supernatants were collected and weighed, and WHC was calculated according to the following Equation (2):

WHC(%)=1-(W1/W2)×100

where *W*1 is the weight of supernatant after centrifugation (*g*), and *W*2 is the weight of the fermented milk before centrifugation (*g*).

### Quantification of Exopolysaccharides Produced in Fermented Milk

The procedure for isolation and purification of free EPS previously reported by Mende et al. (2012) and by Rimada and Abraham (2003) was used. After adding 0.7 ml 80% (*w*/*v*) trichloroacetic acid and heating to 90°C for 15 min, fermented milk samples, when the pH reached the value of 4.5, were cooled in ice water and centrifuged (2,000 rpm, 20 min, 4°C) to remove cells and proteins. After neutralization of the supernatant with NaOH, the EPS were purified following the procedure previously reported (Rimada and Abraham, 2003), and the quantification of the polymers was determined as polymer dry mass (Van Geel-Schutten et al., 1999). Samples were centrifuged (20 min at 2,600 rpm), and the pellets were air dried into an oven at 55°C. Dried EPS were weighted using an Acculab Precision Analytical Balances (Bradford, MA, United States) with four decimal places.

### Statistical Analysis

For each parameter considered, one-way ANOVA and Tukey honestly significant difference *post hoc* tests (α = 0.05) were performed to detect significant differences among the different analysis performed for each strain. Data of each parameter considered were obtained from two independent fermentation processes, and each sample was analyzed in triplicate. The degree of correlation for the parameters measured was checked by means of Pearson’s correlation coefficients (*r*); the correlation was considered significant considering an α = 0.05.

## Results

### EPS Characterization

extracellular polysaccharides produced by *W*4451 has been characterized in terms of monosaccharides relative composition by GC-MS analysis and compared with those produced by the two *Ld. bulgaricus* strains (Table 1). Glucose, rhamnose, and mannose were present to a different extent in the EPS produced by both *Ld. bulgaricus* strains. *Ldb*2214 EPS was characterized by the presence of glucose as the main monosaccharide (90.0 ± 8.0%), while *Ldb*147 EPS was mainly made of galactose (48.2 ± 4.3%) and glucose (42.2 ± 3.8%). Even if in low amount, ribose was detected only in *Ldb*147 EPS. *W*4451 EPS resulted to be an HePS made of ∼50% glucose and rhamnose, with galactose mannose and ribose as the remaining part. Interestingly, *W*4451 EPS residual monosaccharides composition was similar to the EPS from *Ldb*147.

**TABLE 1:** Monosaccharides composition of EPS, reported as relative percentage.

Monosaccharide	Relative percentage of composition and SD		
	*Weissella*	*Lb. delbrueckii* subsp.	*Lb. delbrueckii* subsp.
	4451	*bulgaricus* 2214	*bulgaricus* 147
Rhamnose	12.4 ± 1.1	9.1 ± 0.8	2.8 ± 0.3
Ribose	0.5 ± 0.5	Nd	0.8 ± 0.1
Mannose	12.6 ± 1.1	0.9 ± 0.1	6.0 ± 0.5
Galactose	17.5 ± 1.6	Nd	48.2 ± 4.3
Glucose	57.0 ± 5.1	90.0 ± 8.0	42.2 ± 3.8

*SD, standard deviation; Nd, not detected.*

### Skim Milk Fermentation

To evaluate the effect of EPS on milk rheological characteristics, the EPS+ strains (*W*4451, *Ldb*2214, and *Ldb*147) were cultivated in SSM at 37°C. As a negative control, the EPS− strain (*Ll*220) was incubated in SSM at 30°C.

**FIGURE 1:**
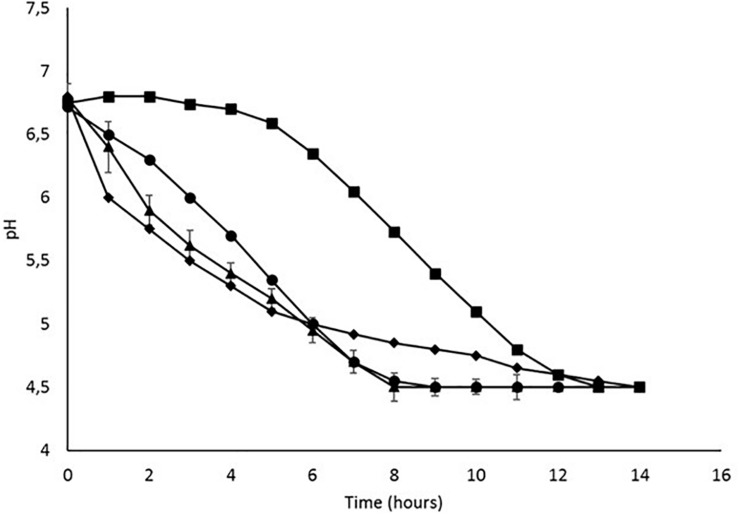
Batch fermentation profile of (●) *Ld. bulgaricus* 2214, (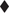) *Ld*. *bulgaricus* 147, (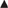) *W. minor* 4451, and (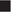) *Lc. lactis* 220, in SSM.

pH was measured over time for all the strains until the value of 4.5 was reached (Figure 1). The acidification time was different depending on the strains: *Ldb147* was initially the faster acidifying strain but reached the pH of 4.5 only after 13 h. *Ldb2214* showed a gradual decrease, reaching pH 4.5 in 9 h, while *W4451* was the faster acidifying strain as it reached pH 4.5 in 8 h. The negative control, *Ll220*, begin to acidify milk after 4–5 h, reaching pH 4.5 only after 12 h.

### Exopolysaccharides Production and Viscosity of Fermented Milks

When pH 4.5 was reached, significantly different concentrations of EPS, varying from 1.88 g/L for *Ldb*2214 to 0.96 g/L for *Ldb*147, were found (Table 2). A very low concentration of polymers dry mass was found also for the EPS− strain *Ll*220, probably as a drawback of the EPS extraction method (Rimada and Abraham, 2003). *W*4451 was able to produce a concentration of EPS in milk of 1.58 g/L, a little smaller than *Ldb*2214.

**TABLE 2:** Exopolysaccharides production (PDM), viscosity (measured by mean of Ford cup and viscometer) and water holding capacity in milk fermented by EPS producers *Weissella* 4451 and *Lactobacillus delbrueckii* subsp. *bulgaricus* 147 and 2214 and negative control EPS-*Lactococcus lactis* 220.

Strains	EPS production	Viscosity				Water holding capacity
		Ford cup	Viscometer			
	PDM g/L	g/min	*n* (−)	*K*(Pa⋅s*^*n*^*)	η_3__.__96_ (Pa⋅s)	WHC (%)
*Weissella* 4451	1.58^b^	0.64^b^	0.578^a^	0.844^b^	0.500^b^	41.59^b^
*Lactobacillus delbrueckii* subsp. *bulgaricus* 147	0.96^c^	1.22^a^	0.353^c^	0.863^b^	0.349^c^	37.72^c^
*Lactobacillus delbrueckii* subsp. *bulgaricus* 2214	1.88^a^	0.34^d^	0.553^ab^	1.182^a^	0.667^a^	51.88^a^
*Lactococcus lactis* 220	0.21^d^	3.24^c^	0.435^bc^	0.492^c^	0.222^d^	35.28^d^

*Data were obtained from two independent fermentation processes, and each sample was analyzed in triplicate. ^*a–d*^Values (mean of the triplicate) followed by different letters within the same column are significantly different (Tukey’s honestly significant difference, *p* < 0.05). PDM, polymer dry mass; *n* (-), flow behavior index; K (Pa⋅s*^*n*^*), consistency index; η_**3.96**_ (Pa⋅s), apparent viscosity; WHC, water holding capacity.*

Considering the high EPS concentration observed for the EPS+ strains, the rheological behavior of fermented milks was measured to evaluate, in particular, the ability of *W*4451 to modify milk’s viscosity in comparison to the other strains considered. The obtained rheological data from flow curves (shear rate vs. shear stress) were well fitted by the power law model, showing high determination coefficients (*R*^2^ = 0.94–0.99). All fermented milks showed a pseudoplastic, shear-thinning behavior (Table 2), as the apparent viscosity strongly decreased with the increase in shear rate (Hess et al., 1997). Values of flow behavior indices (*n*) varied from 0.353 ± 0.112 for *Ldb*147, which was characterized by the most pseudoplastic behavior, to 0.578 ± 0.071 for *W*4451.

*K* coefficient indices, which indicate the consistency of the coagulum at a low shear rate (1 s^–1^), showed significant differences (*p* < 0.05) that were related to the differences in apparent viscosity values (η_3__.__96_) for *Ldb*2214 and *Ll*220; on the contrary, *K* index did not exhibit a significant variation for *Ldb*147 and *W*4451 while having a different η_3__.__96_ value.

This observation can be explained by the different flow behavior between *Ldb*147 and *W*4451 that is observable by the reported η values. However, *W*4451, together with *Ldb*2214, was the best EPS producer and showed also significantly higher values of apparent viscosity of the fermented milk when compared with *Ldb*147 and the EPS− *Ll*220 (+ 30.2 and + 55.6% of η_3__.__96_ values, respectively). In particular, *Ldb*2214 showed the highest value of consistency index and apparent viscosity (*K* = 1.182 Pa⋅s*^*n*^*, η_3__.__96_ = 0.667 Pa⋅s) (*p* < 0.05), resulting in a thicker and firmer structure (Table 2).

With the application of a viscometer, it is possible to measure the dynamic viscosity and the shear-dependent, flow behavior of the fluid; however, this instrument could not be practical and affordable in the case of small or medium-sized dairies. Several faster and internal quality control-oriented methods exist such as Ford cup method. Ford cup is less expensive than a common viscometer; it does not need maintenance or specific extra tools (e.g., specific spindles and cups).

**FIGURE 2:**
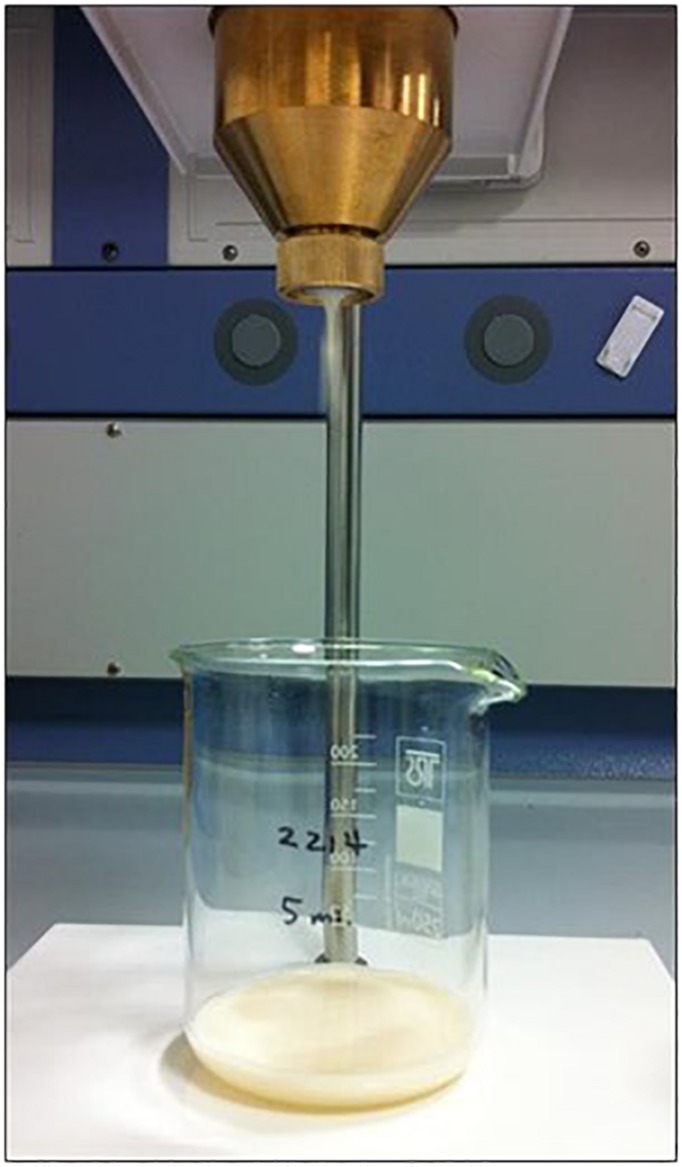
Ford cup used to measure the viscosity of fermented milk by weighing the milk flowing through an orifice of 3 mm diameter.

With the intention to propose an easy and less-expensive method than rotational viscometer to evaluate and monitor milk gelation, the viscosity of fermented milks, expressed as flowrate (g/min), was measured also by weighing the milk flowing through a Ford cup (Han et al., 2016) in a fixed time (Figure 2). Milk fermented by EPS− *Ll*220 showed the highest flowrate, resulting as the less viscous sample, while *W*4451 produced, together with *Ldb*2214, the most viscous sample (Table 2). Ford cup data were in sufficient agreement (*r* = 0.85, *p* < 0.001) with the apparent viscosity measured using the viscometer, allowing the discrimination between the strains (Table 2).

### Water Holding Capacity Measurement

The results of the WHC measurements (Table 2) showed different behaviors between strains EPS+ and EPS−; in particular, the milk fermented by the EPS− strain was significantly more susceptible to syneresis (*p* < 0.05) compared to the EPS+ fermented milk samples. Whey retention was significantly higher (*p* < 0.05) in milk fermented by *W*4451 than by *Ldb*147 (EPS+) and *Ll*220 (EPS−). As expected, the strain *Ldb*2214, which was the best EPS producer, showed the highest (*p* < 0.05) WHC value. The milk fermented by this strain resulted also in more viscosity.

## Discussion

*Weissella minor* strain (*W*4451), analyzed in the present work, isolated from sourdough, was able to ferment SSM by consuming lactose as carbon source and to reach the final pH of 4.5 faster than two dairy isolates *Ldb*2214 and *Ldb*147. The frequent presence of *Weissella* strains in different fermented products suggest their ability to adapt to different environment and different sugars availability (Abriouel et al., 2015). However, to the best of the authors’ knowledge, there are no specific literature data on *W. minor* ability to ferment milk, but at the same time, some data are available about the inability *W. confusa* and *Weissella cibaria* to use lactose and galactose (Quattrini et al., 2019). In our study, when fermentation was stopped, *W4451* was able to produce HePS similarly to other LAB strains such as *Ldb*2214 and *Ldb*147, although the EPS produced were different either for the monosaccharides composition or percentage of the repeating units (Table 1). These results were in agreement with the previously observed structural diversity of EPS produced by LAB (Zeidan et al., 2017), confirming that galactose and glucose, and to a lesser extent mannose and rhamnose, are the most frequently occurring sugars in EPS produced by lactobacilli.

The EPS produced by *W*4451 were characterized by a composition similar to the HePS produced by *W. cibaria* (Adesulu-Dahunsi et al., 2018) but different from other *Weissella* species’ EPS (Bejar et al., 2013; Adesulu-Dahunsi et al., 2018).

When cultivated in SSM, *W*5541 turned out to be the fastest acidifying strain, if compared to *Ll*220 *Ldb*2214, and *Ldb*147, which were previously shown to have variable, strain-dependent, acidification attitudes (Bancalari et al., 2016). This result is in agreement with the work of Fessard and Remize (2017), who found *Weissella* spp. to have an acidifying capacity similar to *Lactobacillus* or *Leuconostoc* in food fermentations. As the pH can affect the viscosity of fermented milks by modifying the structure and the interactions among caseins (Schkoda et al., 1999), the permeability of the gels (Lee and Lucey, 2004), the WHC, and potentially EPS breakage (Pham et al., 2000); the fermentations were stopped at pH 4.5 by rapid cooling at 4°C.

At the end of SSM fermentation (pH 4.5), it was possible to observe differences in the appearance of milks fermented by the EPS+ strains. *W*4451 produced a more compact and uniform clot, similarly to *Ldb*2214. Differently, *Ldb*147 produced a grainier and looser clot similarly to EPS− strain *Ll*220 (data not shown). The different appearance of the clots may be related to the different EPS composition, as previously observed (Vincent et al., 2001).

Significantly different amounts of EPS were present after milk fermentation, varying from 1.88 g/L for *Ldb*2214 to 0.96 g/L for *Ldb*147 (Table 2). On the other hand, it is known that the amount and effect of EPS synthesized *in situ* can vary considerably, depending on the strain, substrate, and fermentation conditions (Mende et al., 2013).

Noteworthy, *W*4451, originally isolated from a non-dairy matrix, produced just a slightly smaller concentration of EPS as compared to *Ldb*2214, one of the species more frequently used to produce yogurt and the majority of fermented milks (Kafsi Hela et al., 2014). Considering that EPS production can be accompanied by a variation in the apparent viscosity of the medium (Mende et al., 2013), the ability of *W*4451 to modify milk’s gel structure and rheology was evaluated and compared to the other strains behaviors.

*W*4451 and *Ldb*2214 strains produced fermented milks with a lower shear rate dependence than the other evaluated LAB strains. The relatively low structured gel behaviors could be related to differences in terms of molecular weight of the produced EPS, as strong differences in terms of EPS composition were not highlighted. In fact, it has been reported that polysaccharides characterized by higher molecular weights can promote a more pronounced shear thinning behavior (Cui et al., 1994; Kafsi Hela et al., 2014).

As previously reported for other LAB (Ayala-Hernández et al., 2009) and *Weissella* species (Benhouna et al., 2019) also EPS produced by *W*4451 and *Ldb*2214 showed good technological and rheological properties that are mainly related to the interaction between milk proteins and polysaccharides. Differently from Benhouna et al. (2019), who used extracted EPS by *W. confusa* as an additive able to improve viscosity of milk gels acidified by glucono delta-lactone, we have demonstrated the ability of a *W. minor* strain to acidify milk and produce EPS *in situ.*

Other authors observed that EPS produced by different LAB strains could be characterized by different composition and structure, and this can greatly influence the ability of EPS to interact with milk proteins and modify rheology of fermented milks, by increasing or decreasing viscosity (Purohit et al., 2009; Sodini et al., 2010). In our study, a different chemical composition of EPS produced by the strains was observed, but this probably influence milk viscosity less than the EPS concentration; in fact, the amount of produced EPS by *W. minor* and the two *Ld. bulgaricus* strains was directly correlated (*r* = 0.93, *p* < 0.001) with the measured apparent viscosity and WHC (*r* = 0.87, *p* < 0.001) of fermented milks (Table 2).

These findings are in agreement with what were reported by other authors, in the case of *W. confusa* (Benhouna et al., 2019) and *Streptococcus thermophilus* (Mende et al., 2013). However, it should be pointed out that other authors observed also a different trend, with viscosity decrease at high concentrations of EPS (Marshall and Rawson, 1999; Sodini et al., 2010). These opposite trends can be justified by molecular differences and structure of EPS (e.g., capsular and ropy EPS, type and density of the charges present on the molecules and their interactions with caseins, etc.) (Purwandari et al., 2007; Corredig et al., 2011), as the texturizing effect is strain dependent and specific (Sodini et al., 2010).

To confirm that the changes in milk rheological properties were mainly caused by the production of EPS, it is important to observe that the EPS− *Ll*220 produced the fermented milk with the lowest apparent viscosity and consistency index, in accordance to previous observations (Hassan et al., 2003).

The best EPS-producing strains showed also the highest WHC values and produced the most viscous fermented milks. These results confirmed that WHC and physical stability are also in relation with the presence and the amount of EPS produced, which improves the stability against whey syneresis (Han et al., 2016).

The ability of *W4451* EPS to limit syneresis in fermented milk is in agreement with recent observation (Purohit et al., 2009; Benhouna et al., 2019), and it is of central importance because the syneresis of yogurt-like products is delayed and limited during shelf-life by EPS interaction with the free water in the gel-like structure (Guzel-Seydim et al., 2005; Okoth et al., 2011).

In conclusion, *W. minor* wild-strain *W*4451, previously isolated from sourdough, was able to acidify milk, produce EPS *in situ*, and increase viscosity of the final product. This ability was comparable to that of two *Ld. bulgaricus* strains, commonly used to acidify milk and to produce EPS. Thus, the wild-strain *W. minor W*4451 may represent a good candidate to be used as starter in milk fermentation. The ability of *W*4451 to modify the viscosity of the gel could be measured also using the Ford cup. The Ford cup turned out to be a useful and simple method to control fermented milk viscosity by small- or medium-sized dairies.

## Data Availability Statement

The datasets generated for this study are available on request to the corresponding author.

## Author Contributions

EB: project design, acquisition, analysis, and interpretation of data, and manuscript drafting. MA: project design, analysis, and data interpretation. BB: manuscript drafting and critical revision. AC: contribution to the chemical analysis. GM: critical revision and finalization of the manuscript. MG: data interpretation, critical revision, and finalization of the manuscript.

## Conflict of Interest

The authors declare that the research was conducted in the absence of any commercial or financial relationships that could be construed as a potential conflict of interest.
